# Long-Term Impact of Phosphorous Fertilization on Yield and Alternate Bearing in Intensive Irrigated Olive Cultivation

**DOI:** 10.3390/plants10091821

**Published:** 2021-09-01

**Authors:** Amnon Haberman, Arnon Dag, Ran Erel, Isaac Zipori, Nerya Shtern, Alon Ben-Gal, Uri Yermiyahu

**Affiliations:** 1 Gilat Research Center, Agricultural Research Organization, M.P. Negev 85280, Gilat, Israel; amnon.haberman@csiro.au (A.H.); arnondag@volcani.agri.gov.il (A.D.); ranerel@volcani.agri.gov.il (R.E.); matabsor@volcani.agri.gov.il (I.Z.); nerya6040@gmail.com (N.S.); bengal@volcani.agri.gov.il (A.B.-G.); 2 CSIRO Agriculture and Food, Waite Campus, Glen Osmond, SA 5064, Australia

**Keywords:** *Olea europaea*, fertigation, fruit set, flowering, mineral nutrition

## Abstract

Phosphorus (P) availability significantly impacts olive tree reproductive development and consequential fruit production. However, the importance of P fertilization in olive cultivation is not clear, and P application is usually recommended only after P deficiency is identified. In order to determine the long-term impacts of continuous P fertilization in intensive irrigated olive cultivation, the growth and production of trees in an intensive orchard with or without P fertilization were evaluated over six consecutive seasons. Withholding of P resulted in significant reduction in soil P quantity and availability. Under lower P availability, long-term fruit production was significantly impaired due to reduced flowering and fruit set. In addition, trees under conditions of low P were characterized by higher alternate bearing fluctuations. Olive tree vegetative growth was hardly affected by P fertilizer level. The impairment of tree productivity was evident in spite of the fact that leaf P content in the treatment without P fertilization did not decrease below commonly reported and accepted thresholds for P deficiency. This implies that the leaf P content sufficiency threshold for intensive olive orchards should be reconsidered. The results demonstrate the negative impact of insufficient P fertilization and signify the need for routine P fertilization in intensive olive cultivation.

## 1. Introduction

Phosphorus (P) is an essential plant macronutrient, participating in an array of plant functions [1,2]. Plants generally absorb orthophosphate through their roots from the soil solution. Most soils are rich in P, which can exist in both inorganic and organic form [3]. The vast majority of the inorganic P is firmly fixed in the soil due to its precipitation with calcium (Ca) or adsorption by iron (Fe) and aluminum (Al) oxides [4]. Only a small fraction of the soil inorganic P is therefore available for plant uptake, and P release rates from soil minerals are generally too slow to meet the crop demand [3]. Most soil organic P is found in stable organic structures, making it also hardly available for plant uptake [5]. Plants can accelerate organic P mineralization by releasing acidic phosphatases from roots and by affecting the rhizosphere chemical conditions, resulting in a higher release of inorganic P to the soil solution, where it becomes available for plant uptake [4,6].

Due to the restricted levels of plant-available P in many arable soils, P deficiency is commonly estimated to limit crop production [7,8]. Hence, to better fulfill crop demand and support higher production, P is typically added to the soil or by foliar spray in agricultural systems. Phosphorus fertilizers are rapidly fixed to the soil, with a labile P portion available for plant roots. Fertilizer application via the irrigation system (fertigation) has been shown to enhance P availability momentarily [9,10,11], leading to a significant increase in P utilization efficiency [12] and P mobility in the soil profile [13]. As a result, P availability in fertigation is superior to solid fertilizer application, stemming from the higher lifespan of the orthophosphate in the soil solution [14,15].

Phosphorus application in olive (*Olea europaea* L.) cultivation is often considered unnecessary and is recommended only when apparent deficiencies, usually according to leaf P content, are detected. A commonly accepted threshold for P deficiency in leaves is 0.1% [16,17,18,19]. This approach is accepted due to the rarity of apparent P deficiency symptoms [17,20] and the presumption that, due to their extensive root system and symbiosis with mycorrhiza fungi [21,22], olive tree P uptake is very efficient [16,18,23]. In addition, several studies failed to find conclusive evidence to support the benefits of routine P application in olive cultivation [24,25,26,27,28].

Over the last decades, the olive sector has transitioned from traditional, rain-fed, low-intensity to intensive irrigated cultivation. Modern intensive olive orchards are planted with new cultivars at higher planting densities on fertile soils. In addition, innovative agricultural practices, such as micro-irrigation systems and fertigation technologies [10], have been integrated into orchard management. Consequently, production levels per area in modern intensive orchards are substantially higher than in more traditional orchards [29,30]. It is likely that the P requirements of intensive olive orchards differ from those of more traditional orchards. On the one hand, P demand would probably increase due to the higher density, growth rates, and production. On the other hand, the integration of irrigation is expected to impact the availability of otherwise immobile plant nutrients in the soil [31], such as calcium, potassium, and phosphorus.

Recent studies on olive tree mineral nutrition, using soilless potted experimental systems, revealed that higher P availability had a significant positive impact on fruit production [32,33,34]. The increased production was attributed to higher flowering intensity, percent of perfect flowers, pistil weight, fruit set, and fruitlet persistence, all resulting from higher P nutritional levels. In addition, carbon assimilation rate and stomatal conductance were reduced in P deficient trees [34].

The benefits of routine P application in commercial olive cultivation are still questionable [16,18,35]. However, low P availability was found to restrict reproductive growth and yield of potted olive trees [34], suggesting that, in intensive cultivation, where growth and yield are substantially increased [36] and water availability is no longer a dominant limiting factor, P application may support higher production, even if trees are not apparently P deficient in terms of leaf content. In order to determine the long-term impacts of continuous P fertilization in intensive irrigated olive cultivation, we evaluated the growth and production of trees growing in an intensive orchard with or without P fertilization over six consecutive seasons. The long-term impacts of P fertilization on P availability in the soil and tree growth and production are presented and discussed.

## 2. Results

### 2.1. Soil P Content

**Figure 1:**
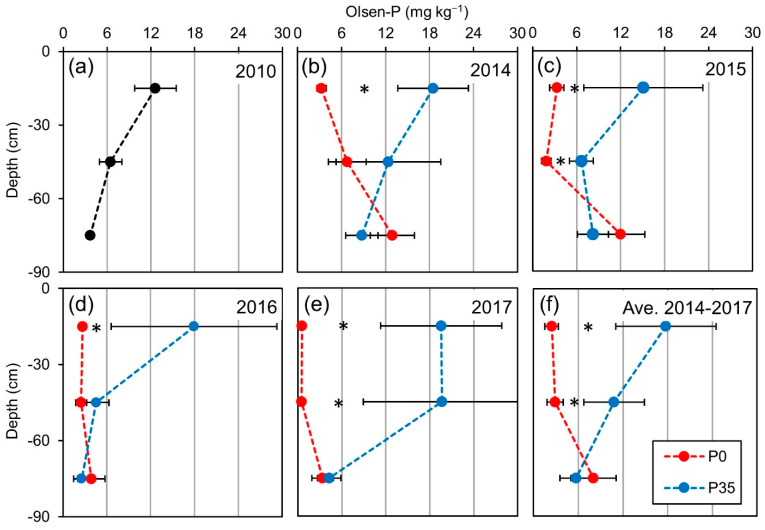
Olsen-P content in soil profile throughout the experiment. Experiment plots were fertilized either with an annual amount of 35 kg P ha^−1^ (P35) or without P (P0). The Olsen-P was measured for three soil depths (0–30, 30–60, 60–90 cm) in February 2010 (before the initiation of the treatments; (**a**), March 2014 (**b**), 2015 (**c**), 2016 (**d**), 2017 (**e**) and calculated average of 2014–2017 (**f**). Numbers are mean values of 6–7 replicates (plots) ± standard error of the means (bars). Asterisks indicate a significant statistical difference between fertilization levels in the specified depth (*p* ≤ 0.05).

Soil extractable (Olsen) P, following three seasons without P fertilization (P0), decreased significantly in the upper soil profile (0–30 cm; Figure 1a,b). This trend intensified during the experiment, leading to lower availability of P to the trees in subsequent years. After six consecutive seasons of no P fertilization, the Olsen-P content diminished to very low levels in the upper soil profile and also in the deeper soil profile (Figure 1e). In contrast, under regular P fertilization (P35), Olsen-P content somewhat increased compared to the before the initiation of the treatments, predominantly in the upper soil (Figure 1). The differential fertilization hardly affected the lower soil profile (Figure 1f), as expected due to the low mobility of P in the soil.

**Figure 2:**
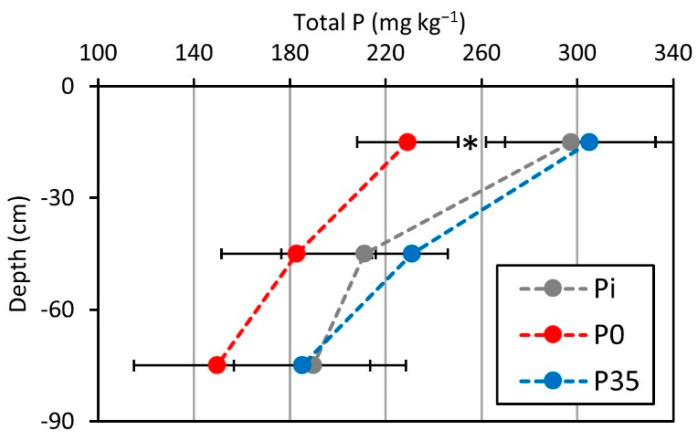
Total P content in soil profiles. Experiment plots were fertilized either with an annual amount of 35 kg P ha^−1^ (P35) or without P (P0). The soil total P content was measured for three soil depths (0–30, 30–60, 60–90 cm) in March 2017 after six seasons of differential fertilization or in February 2010 before the experiment (Pi). Numbers are mean values of 7–8 replicates (plots) ± standard error of the mean (bars). Asterisks indicate a significant statistical difference between fertilization levels in the specified depth (*p* ≤ 0.05).

Changes in total P levels reflect the net accumulation or depletion of P within the soil profile, regardless of the P form and bioavailability. Similar to the Olsen-P levels, following six seasons of denying P fertilization (P0), total P in the soil diminished substantially compared to initial levels (Pi; Figure 2). However, the depletion of total P during the experiment was statically significant only in the upper soil layer. Contrarily, in the P fertilized plots (P35), the total P content in soil was not significantly affected (Figure 2).

### 2.2. Leaf and Fruit Macronutrient Content

**Figure 3:**
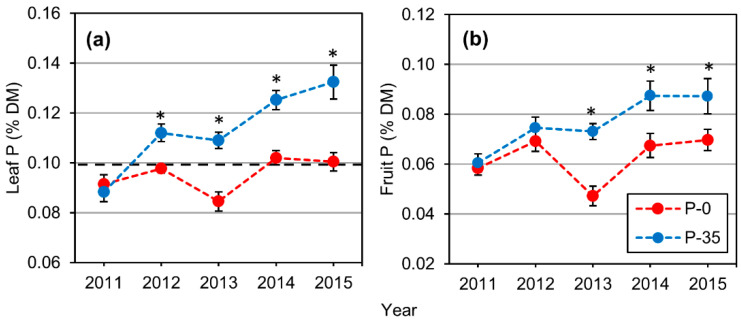
Phosphorus in leaves and fruit. Experiment plots were fertilized either with an annual amount of 35 kg P ha^−1^ (P35) or without P (P0). (**a**) Leaf P content. (**b**) Fruit P content at harvest as percent of dry mass (DM). Numbers are mean values of 14 replicates (trees) ± standard error of the mean (bars). The horizontal dashed line indicates the accepted threshold for adequate P content in leaves. Asterisks indicate a significant statistical difference between P fertilization levels in the specified season (*p* ≤ 0.05).

At the beginning of the experiment, the leaf P content was relatively low (Figure 3a), slightly under the 0.1% threshold level proposed in the literature to be adequate [16,18]. Despite the absence of P fertilization for a prolonged period (five years), the leaf P content did not decrease (Figure 3a). In contrast, leaf P content in the P fertilization treatment increased significantly after only one season and continued to rise through the experiment (Figure 3a). Leaf N and K content were not affected by the P fertilization (Figure S1). Trends in fruit P content over time were similar to those in the leaves (Figure 3). Fruit P content significantly increased under continuous P fertilization, particularly in the later seasons of the experiment (2014–2015; Figure 3b). The P content in fruit from trees fertilized without P was not significantly reduced, with the exception of in the 2013 season when both fruit and leaf P content in both treatments were particularly low without apparent reason (Figure 3).

### 2.3. Vegetative Growth

Statistically insignificant slight increases were found in tree trunk circumference and annual pruning weight in trees fertilized without P (Table 1). It appears that P availability did not have a substantial impact on tree vegetative growth.

### 2.4. Flowering and Fruit Set

In contrast to vegetative development, reproductive developmental parameters were affected significantly by P availability. Both flowering intensity and inflorescence initiation rate were significantly lower in the P0 treatment (Table 1), indicating that P fertilization affected tree reproductive development. In addition, the percentage of perfect flowers in the P0 treatment was reduced (Table 2), showing an impact on flowering quality. The effect on the number of flowers in the inflorescence and the flower pistil weight was not significant (Table 2). The fertilization also affected the fruit set rate, which was substantially reduced in the trees fertilized without P (Table 2).

### 2.5. Yields

**Figure 4:**
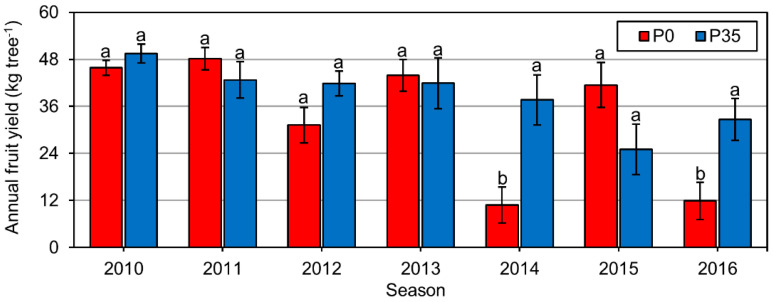
Seasonal fruit yields. Experiment plots were fertilized either with an annual amount of 35 P ha^−1^ (P35) or without P (P0). Numbers are mean values of 14 replicates (trees) ± standard error of the mean (bars). Different letters indicate a statistically significant difference between P fertilization levels in the specified season (*p* ≤ 0.05).

The P fertilization did not influence fruit characteristics. Single fruit weight and oil content were hardly affected (Table 3). Nevertheless, the absence of P fertilization resulted in a significant reduction of 20% in fruit yield. This reduction did not occur as a linear trend (Figure 4) due to the dominance of alternate bearing in controlling yield levels [39]. To normalize the effect of alternate bearing, the impact on yield was evaluated for the mean level of the four later seasons (2013–2016) of the experiment. Yields from the experiment’s early seasons (2011–2012) were omitted to overcome delays in the treatment effects due to the potential impact of P reserves in and on the trees and the soil. The reduced fruit yield resulted in a significant reduction of 18% in the oil yield (Table 3), both of which can be attributed to the decrease in the number of fruit produced per tree (Table 3).

### 2.6. Alternate Bearing

Following an on-year (high crop load season), flowering intensity in trees receiving the P0 treatment was substantively reduced (Table 4), resulting in a more severe tendency to alternate bearing. The calculated alternate bearing intensity index for the trees fertilized without P was significantly higher (Table 4), resulting from higher crop fluctuations. Also, it appears that the trees in the P0 treatment were more synchronized regarding on/off-years crop fluctuations (Figure 4) and that the phenomenon of alternate bearing increased in the absence of P fertilization.

## 3. Discussion

### 3.1. Soil P Content

Extractable inorganic P levels in the soil, commonly determined in alkaline soils using the Olsen method [40], are considered to provide reasonable estimates for plant-available P. However, Olsen-P measurements occasionally fail to explain plant P uptake [41,42,43]. The relationships between extractable (Olsen) and plant-available P are dependent on soil properties and P forms [41,42]. Hence, identifying adequate extractable P thresholds that support sufficient P plant-uptake is challenging. Nevertheless, changes in Olsen-P values can represent general trends in immediate plant-available P levels. The rapid depletion of extractable P in the upper soil in the current experiment demonstrates that P availability can decrease significantly without fertilization, which is the common practice in many olive orchards [19]. The lower response deeper in the profile is not surprising, as P mobility in alkaline and clayey soils is poor [3].

Chatzistathis et al. [35] recently claimed that olive tree P nutritional needs could be satisfied by the soil organic matter. This probably applies specifically to high organic matter soils, like that in their study having 4.15% organic matter in the 0–30 cm soil horizon. Olive orchards are typically planted on sloping lands with relatively low soil fertility [44], which in some cases means lower organic matter content. In the current study, the organic matter content in the upper soil (0–30 cm) was 0.56%, typical of soils used for olive cultivation in Israel and the Mediterranean Basin [45]. The substantial difference in organic matter content between the soils in the [35] study and ours may explain the different conclusions regarding the significance of P fertilization.

The reduction in soil total P throughout the experiment in the upper soil layer (0–30 cm) of unfertilized plots was 68.1 mg kg^−1^ (calculated from the data presented in Figure 2), representing 23% of the initial soil P content. This significant depletion in total soil P, together with the substantial decrease in extractable P, illustrates that not fertilizing with P is not sustainable for the given agro-system. Annual application of 35 kg ha^−1^ of P fertilizer was associated with a somewhat elevated level of extractable P (Figure 2), a slight statistically insignificant increase in total soil P (Figure 2), and a gradual increase in leaf P to 0.13% (Figure 3). This increase in soil and plant P indicators implies that the annual application rate of 35 kg ha^−1^ may have been somewhat higher than locally required, consequently leading to P accumulation in the soil and trees.

### 3.2. Leaf P Content and Thresholds

Leaf P content in trees fertilized with P increased and was significantly higher, indicating that the elevated soil P availability promoted P uptake by the roots. However, P leaf content in trees fertilized without P did not decrease despite the P depletion in the soil. It appears that tree roots continued to supply P to sustain levels even under the extremely low soil P. Zipori et al. [31] reported that increasing irrigation rates resulted in higher P leaf content. The experimental orchard was well irrigated, possibly facilitating P desorption from soil minerals to the soil solution and relatively high and prolonged concentrations of P dissolved in solution available for root uptake [13]. This postulation is supported by the marked reduction in the soil total P while extractable P levels were low. In addition, P levels may have been sustained by increased remobilization and recycling of P within the tree.

Leaf P content strongly and linearly relates to olive fruit production [33]. Critical deficiency levels for leaf P content published in olive production guides are around 0.05–0.07%, and 0.1% is given as a general sufficiency thresholds [16,18]. The lowest leaf P content observed in the P0 treatment was 0.085%, while in most of the experiment duration, levels were about 0.1%. Thus, leaf diagnostics did not indicate P deficiency according to published thresholds. The fact that decreased P availability negatively impacted fruit production without lowering leaf P content below 0.1% implies that the commonly published sufficiency/deficiency thresholds may be under-estimated, at least for the conditions of the experiment. We suggest that leaf P content sufficiency threshold in intensive irrigated cultivation may be above 0.12%, as already practiced by olive growers in Israel [19].

### 3.3. Vegetative and Reproductive Growth

Phosphorus is involved in many physiological processes and also plays a key role in cell energy transfer [2]. Under P deficiency, tree physiology may not perform at the optimal level, leading to a general lower tree fitness. Phosphorus deficient olive trees presented reduced carbon assimilation rate and stomatal conductance [34], which may result in lower resource availability to support growth. In addition, when trees are starved for P, more carbon is allocated for root growth to enhance P exploration [46]. Therefore, because of the high energetic investment in the root system, fewer resources might be available for above-ground development [47].

Reports from potted tree systems suggest that P availability can impact tree vigor. Trunk circumference of potted olive trees growing under very low P availability, with deficient leaf P content (0.06%), was significantly smaller [33,34]. In a different study with young potted olive trees, shoot growth was greatly affected by P availability [20]. In the current study, tree vegetative development was hardly affected by the decrease in P availability. For comparison, reduced N availability in a similar experimental system significantly impacted vegetative vigor [38].

In the current study, the flowering of the trees growing without P fertilization was reduced, possibly due to lower general tree fitness and resource availability. Similar trends of impaired flowering were also reported for the impact of N and K availability in a similar experimental system [37,38], suggesting that the down-regulation of flowering is not a direct effect of the specific nutritional deficiency. Pistil abortion, which results in an imperfect flower (andromonoecy), is considered a regulatory pathway adjusting reproduction potential to resource availability [48,49,50]. Pistil abortion rates in olive shoots were augmented by reducing the leaf area to buds ratio [51]. Thus, reduced photosynthetic capacity may promote a lower rate of perfect flowers. This is also supported by the correlation found between pistil abortion and the pistil starch content [52]. The reduced rate of perfect flowers in the P0 treatment may testify to the reduction in tree fitness under P deficiency. The impact of P availability on the rate of perfect flowers was also found in trials with potted olive trees [33,34], and in other species such as pomegranate (*Punica granatum*) [53].

Higher flower pistil weight was reported to associate with a higher fruit set [54]. Erel et al. [34] found that pistil weight positively correlated to increasing P availability. The pistil weight in the current study was only slightly reduced in the P0 treatment, without statistical significance. Nevertheless, reduced P availability did impact the eventual rate of fruit set, similar to previous reports for P in potted olive trees [32,55]. The combined effect of the reduced flowering and fruit set under lack of P fertilization explains the 20% reduction in fruit production.

### 3.4. Yields

Overall, we report that routine P fertilization promoted higher yields, unlike previous studies that did not find a significant response [24,25,26,27,28]. Our contrasting findings are probably a result of the different agro-system in question. In modern irrigated high-density systems, growth and yields are substantially higher than in traditional rainfed settings, resulting in higher P requirements. Thus, the positive impact of P fertilization is probably due to increased demand under routine irrigation. This is supported by the finding that increasing irrigation rates resulted in higher leaf P content [31]. Similarly, P fertilization did not increase yields in unirrigated Mung bean (*Vigna radiata* L.) cultivation, whereas in irrigated plots, yields increased in response to P fertilization [56]. Apparently, as long as water availability is the main limiting factor, P fertilization may be ineffective. However, once this limitation is removed, other limiting factors will determine the yield. Phosphorus availability may become a limiting factor in higher demand settings.

The current price of P fertilizer in Israel is about $2.5 per one kg of P_2_O_5_ [57]. At this price, an annual application of 35 kg P ha^−1^ would cost about $200 ha^−1^. This cost should be even lower if using phosphoric acid. In the later seasons of the experiment (2013–2016), the P application promoted an average annual increase in oil yield of 360 kg ha^−1^ (calculated with the data in Table 3 and the orchard density). Assuming that olive oil prices range around $4 kg^−1^ [58], the increase in production translates to roughly $1360 ha^−1^ annually, more than justifying the spending on the P fertilizer.

### 3.5. Alternate Bearing

Alternate bearing is an undesirable horticultural trait of many perennial crops, including olive [39]. Alternate bearing yield fluctuations in olive are driven mainly by the negative feedback of fruit load on vegetative vigor and flowering [59,60]. Thus, promoting vegetative growth and flowering during a high fruit load season (on-year) is ultimately the way to reduce the alternate bearing amplitude without reducing overall yields. In the current study, trees growing under lower P availability demonstrated higher alternate bearing behavior. This can be attributed to the reduced flowering level in these trees and, in particular, to the flowering following a high fruit load season. Haberman et al. [59] previously demonstrated that olive flower induction is mediated by the levels of olive *FT* (*OeFT1/2*) and *TFL1* (*OeTFL1-1*) genes. Perhaps, lower general tree fitness and resource availability affected the expression levels of *OeFT1/2* and *TFL1 OeTFL1-1* to promote down-regulation of floral induction. Bustan et al. [61] reported that the leaf P content was significantly lower in on-year trees, suggesting that P demand may be elevated under high fruit load. Fertilization amounts should therefore perhaps be higher during on-years, in order to meet the higher demand.

## 4. Materials and Methods

### 4.1. Experimental Site

The trial was conducted in a mature commercial orchard situated in Israel’s southern coastal plain (31°39′7.50″ N 34°40′54.00″ E). Soil texture varied from loam to clay loam, soil properties are presented as supplementary material (Table S9). The orchard was planted in 2007 at a density of 360 trees ha^−1^ (4 m × 7 m) with Barnea olive trees. Barnea is a modern variety characterized by erect growth, high yields, and suitable for mechanical harvest [62]. As a result of its vigorous growth, high productivity, and tolerance to salinity [63,64], the ‘Barnea’ gained popularity for oil production in many modern agrosystems globally. In the 2013 season, the first season of the experiments in which tree performances were analyzed, the orchard was six years from planting, and trees reached maximum size. Crop production levels were normal for intensive cultivation in the region.

Trees were irrigated throughout the dry season (March to October) twice a week with fresh-water (EC = 0.4–0.5 dS m^−1^) using drip irrigation. The irrigation system consisted of one 20 mm diameter lateral line per row of trees with 1.6 l h^−1^ flow drippers spaced every 75 cm. Irrigation rates were calculated according to potential evapotranspiration, using a modified Penman-Monteith method [65] and a crop coefficient ranging between 0.27–0.70 depending on the trees’ phenological stage. Annual irrigation rates in the seasons of the trial amounted to 470–630 mm. At the same time, the annual winter precipitation amounted to 280–600 mm (average 460 mm). Meteorological data (rainfall and temperature) for the trial period is presented as supplementary material (Figure S2).

Fertilizers were applied continuously via the drip irrigation system (fertigation) throughout most of the irrigation season. Fertigation was practiced from the beginning of the irrigation season, in each irrigation, and until the annual fertilizer program was complete, at about the end of August. Hence, the fertilizer distribution rate depended on the irrigation rate. Fertilizer solutions, supplied by Israel Chemicals Ltd. (ICL), were compiled to deliver annual application of 150 kg nitrogen (N), 35 kg P (or none), and 250 kg potassium (K) per ha, using ammonium nitrate, phosphoric acid, and potassium chloride. In accordance, average N, P, and K concentrations in the irrigation water, which originated from the added fertilizers, were mostly constant at approximately 30, 7 or 0, and 50 mg l^−1^, respectively. Micronutrients were applied similarly to the macronutrients, at annual doses of 3.8 kg iron (Fe), 1.9 kg zinc (Zn), 940 g manganese (Mn), 140 g copper (Cu), and 100 g molybdenum (Mo) per ha. Application rates were set according to the standard commercial practice for an intensive mature olive orchard in Israel [66].

### 4.2. Experimental Design

The experiment was conducted according to randomized block design. Three ha of the orchard were divided into seven experimental blocks. In each block, plots of 12 uniform trees (Four trees along the row × three rows) were assigned for the different treatments. The two trees in the plot center were assessed, generating 14 surveyed trees per treatment level (biological replications). Two P fertilization treatments were evaluated: annual application of 35 kg P ha^−1^ (P35) and no additional P application (P0). Treatments were initialized in June 2011 and continued each irrigation season through 2016.

### 4.3. Measurements

#### 4.3.1. Soil Analysis

Soil samples were collected from the center of each experimental plot, 5–10 cm from the drip irrigation line, between two adjacent drippers, from three depths: 0–30 cm, 30–60 cm, and 60–90 cm during spring (March) before the beginning of each irrigation season. Baseline soil nutrient content was measured in February 2010, and the final content was measured in March 2017. Soil extractable P content was determined using the Olsen method (extraction with sodium bicarbonate) [40]. Soil total P was extracted with aqua-regia reagent in a microwave system (MARS 6, CEM Corporation, Matthews, NC, USA) and determined by ICP-OES (Agilent 5100, Agilent Technologies, Santa Clara, CA, USA).

#### 4.3.2. Leaf and Fruit Macronutrient Content

Each July, samples of about 100 fully expanded leaves from the current season’s growth of shoots without fruit (diagnostic leaves) were collected from each measured tree. The leaf samples were rinsed in deionised water, oven-dried (70 °C), ground to a powder, and 0.1 g were digested with sulfuric acid and hydrogen peroxide (Merck, Darmstadt, Germany) [67]. The P and N content was determined by colorimetric analysis (Gallery Plus Automated Photometric Analyzer, Thermo Scientific, Waltham, MA, USA). The K content was determined using an atomic absorption spectrometer (AAnalyst 200, Perkin-Elmer, Waltham, MA, USA). Fruit samples collected at harvest were crushed to paste and handled similar to the leaf samples.

#### 4.3.3. Vegetative Growth

Following harvest (January–February), trees were pruned to approximately the same canopy size according to the commercial practice by a professional pruning crew. The pruned material from each tree was weighed separately. Each tree’s trunk circumference, 50 cm above ground, was measured every spring, and the relative increase in the circumference was calculated.

#### 4.3.4. Flowering and Fruit Set

Flowering intensity was estimated visually at full bloom. Trees were given a score on a scale of 0–5, with 5 representing the highest intensity. Additionally, the rate of inflorescence initiation was quantified in the 2014 and 2015 seasons. Before inflorescence initiation (December), six shoots were tagged in each tree. The rate of buds that initiated an inflorescence in the selected shoots was determined during flowering.

Flowering quality traits such as the number of flowers per inflorescence, rate of perfect flowers, and weight of the flower pistil were evaluated using a sample of ten inflorescences from each measured tree. The rate of fruit set was estimated in six pre-selected shoots in each tree. The number of inflorescences in each shoot was recorded at flowering and, three months later, the number of fruit in the shoots was counted. The fruit set rate (the number of fruit per initial number of flowers) was calculated using the mean number of flowers in an inflorescence.

#### 4.3.5. Yields

Trees were harvested using a mechanical trunk-shaker assisted by rod beating. Fruit yield of each tree was weighed separately. The harvest was scheduled to when the fruit color shift from green to purple was about 50%. A three kg fruit sample was taken from each tree, and a sub-sample of 100 fruit was used to determine the average weight of a single fruit. Fruit oil content was determined with one kg sub-samples that were crushed to paste in a laboratory mill (Abencor System; mc2, Ingenieria y Sistemas, Seville, Spain) and evaluated by calibrated near-infrared analysis [68] (OliveScan, Foss, Hilleroed, Denmark).

### 4.4. Data Analysis

Statistical significance was determined according to one-way ANOVA using the Student’s *t*-test or Wilcoxon test for comparisons with unequal variance (*p* ≤ 0.05). To overcome the potential impact of P reserves in the trees and the soil, which may cause delays in the treatment effects, data regarding responses in flowering intensity, flowering quality traits, yield, and alternate bearing from the early seasons (2011–2012) are omitted from the analysis. Alternate bearing impacts are normalized by calculating means with data of two or four consecutive seasons. Seasonal data is presented as supplementary material (Tables S1–S8). The effects of flowering intensity on flowering quality traits were neutralized by excluding trees flowering at very low intensity (0–2) from the analyses of these parameters. The alternate bearing index [69] was calculated per individual tree by subtracting the seasonal yield of two consecutive seasons and then dividing the absolute value obtained by the yield sum in the two seasons. The values obtained for each pair of seasons throughout 2013–2016 seasons were added and divided by the number of observed seasons minus one (4 − 1 = 3).

## 5. Conclusions

The majority of knowledge-based practices for olive management were developed for traditional low-intensity rain-fed orchards. It is reasonable to assume that under intensive cultivation, P demand will increase. Withholding P fertilization for six consecutive seasons in an intensive orchard resulted in depletion of soil P and impaired fruit production. The reduction in tree performance is attributed mostly to reduced flowering and fruit set. In contrast, the P deficiency level hardy affected vegetative vigor. The impaired tree performance was not accompanied by reductions in leaf P content below those commonly accepted as thresholds for deficient P, suggesting that: (1) routine P fertilization in intensive olive cultivation should be considered even without identifying apparent deficiency in leaf diagnostic and (2) the leaf P content threshold(s) dividing deficiency and sufficiency should be reconsidered for intensive irrigated olive cultivation.

## Figures and Tables

**Table 1:** Phosphorus fertilization impact on vegetative and reproductive development.

P Fertilization	Trunk Circumference Increase	Pruning Weight	Flowering Intensity	Inflorescences Initiation ^a^
	2011–2017	2012–2015	2014–2017	2014–2015
(kg ha^−1^ season^−1^)	(%)	(kg tree^−1^ season^−1^)	(index)	(%)
0	69.5 ± 3.0 a	31.1 ± 1.9 a	3.05 ± 0.07 b	36.3 ± 2.4 b
35 ^b^	74.3 ± 2.8 a	34.0 ± 2.5 a	3.34 ± 0.11 a	44.5 ± 3.2 a

Numbers are mean values of 14 replicates (trees) ± standard error of the mean. Different letters indicate a statistically significant difference (*p* ≤ 0.05). ^a^ The rate of buds initiating an inflorescence. ^b^ Values of this level were published previously [37,38]. Seasonal pruning weight, flowering intensity, and inflorescences initiation data are presented in Tables S1–S3, respectively.

**Table 2:** Phosphorus fertilization impact on flowering quality traits and fruit set.

P Fertilization	Flowers in an Inflorescence	Perfect Flowers	Pistil Weight	Fruit Set
(kg ha^−1^ season^−1^)	(No.)	(%)	(mg)	(%)
0	16.1 ± 0.3 a	46.9 ± 3.6 b	0.72 ± 0.02 a	4.5 ± 0.4 b
35 ^a^	16.2 ± 0.5 a	56.5 ± 3.3 a	0.79 ± 0.04 a	6.7 ± 0.6 a

Data from trees flowering at medium to high intensities (index of 3–5) during the 2014–2017 seasons. Numbers are mean values of 27–28 replicates (trees) ± standard error of the mean. Different letters indicate a statistically significant difference (*p* ≤ 0.05). ^a^ Values of this level were published previously [37,38].

**Table 3:** Phosphorus fertilization impact on yields.

P Fertilization	Single Fruit Weight	Oil Content	Fruit per Tree	Fruit Yield	Oil Yield
	2013–2016	2013–2016	2013–2016	2013–2016	2013–2016
(kg ha^−1^ season^−1^)	(g)	(%)	(No. tree^−1^ season^−1^)	(kg tree^−1^ season^−1^)	(kg tree^−1^ season^−1^)
0	2.66 ± 0.11 a	19.2 ± 0.5 a	14,153 ± 825 b	27.2 ± 1.1 b	4.6 ± 0.2 b
35 ^a^	2.42 ± 0.09 a	19.6 ± 0.6 a	19,813 ± 1141 a	34.0 ± 1.6 a	5.6 ± 0.4 a

Numbers are mean values of 14 replicates (trees) ± standard error of the mean. Different letters indicate a statistically significant difference (*p* ≤ 0.05). ^a^ Values of this level were published previously [37,38]. Seasonal single fruit weight, oil content, fruit per tree, fruit yield, and oil yield data are presented in Tables S4–S8, respectively.

**Table 4:** Phosphorus fertilization impact on alternate bearing.

P Fertilization	On-Year Fruit Yield ^a^	Flowering Intensity Following an On-Year ^b^	Alternate Bearing Intensity ^c^
(kg ha^−1^ season^−1^)	(kg tree^−1^)	(index)	(index)
0	44.3 ± 2.0 a	1.6 ± 0.30 b	0.78 ± 0.07 a
35	41.8 ± 2.1 a	3.0 ± 0.25 a	0.59 ± 0.09 b

Numbers are mean values ± standard error of the mean. Different letters indicate a statistically significant difference (*p* ≤ 0.05). ^a^ Mean fruit yield of on-year trees (20–60 kg tree^−1^ in 2013–2016 seasons). ^b^ Following season flowering of the on-year trees. *n*= 25–30 (trees). ^c^ Calculated for 2013–2016 seasons, according to Monselise and Goldschmidt (1982). *n* = 11–12 (trees).

## Data Availability

Data is contained within the article and supplementary material.

## Supplementary Materials

The following are available online at https://www.mdpi.com/article/10.3390/plants10091821/s1, Figure S1: Effect of P fertilization on leaf N and K content, Figure S2: Meteorological data, Table S1: Seasonal pruning weight, Table S2: Seasonal flowering intensity, Table S3: Seasonal inflorescences initiation rate, Table S4: Seasonal single fruit weight, Table S5: Seasonal fruit oil content, Table S6: Seasonal number of fruit per tree, Table S7: Seasonal fruit yield, Table S8: Seasonal estimated oil yields, Table S9: Soil physical and chemical properties.
