# Supplementary material for: Long-Term Impact of Phosphorous Fertilization on Yield and Alternate Bearing in Intensive Irrigated Olive Cultivation
Source: Plants (Basel). 2021 Sep 1;10(9):1821. doi: 10.3390/plants10091821 (PMC8467881; doi:10.3390/plants10091821)
Supplement: Supplementary file 1 [file plants-10-01821-s001.zip › Figure S2_Meteorological Data.pdf]

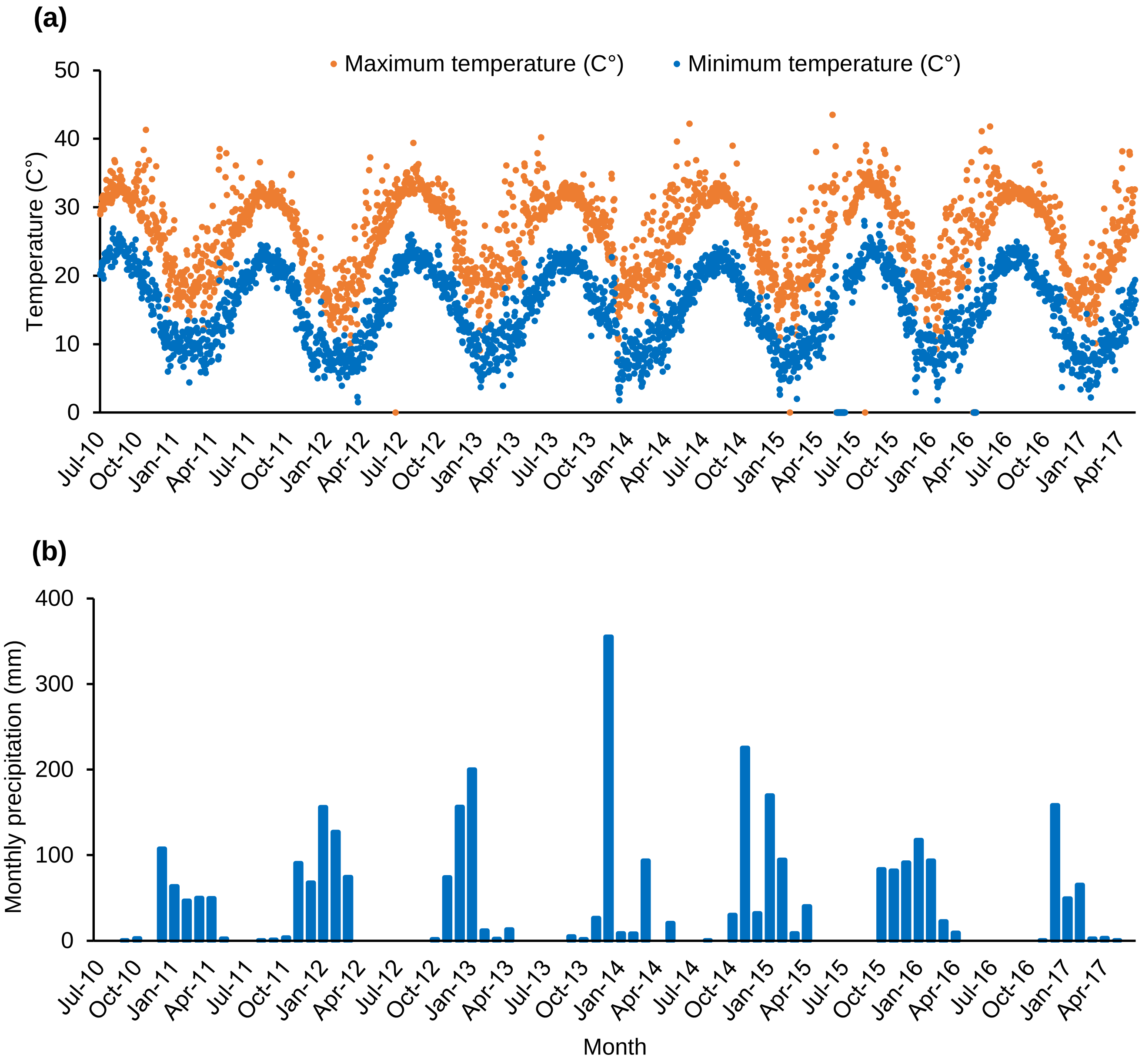

Figure S2: **Meteorological data**. Daily maximum and minimum temperature (a) and monthly precipitation (b) were obtained from a nearby meteorological station (Negba) of the Israeli Ministry of Agriculture and Rural Development.
