# Supplementary material for: Long-Term Impact of Phosphorous Fertilization on Yield and Alternate Bearing in Intensive Irrigated Olive Cultivation
Source: Plants (Basel). 2021 Sep 1;10(9):1821. doi: 10.3390/plants10091821 (PMC8467881; doi:10.3390/plants10091821)
Supplement: Supplementary file 1 [file plants-10-01821-s001.zip › Table S9.pdf]

Table S9: Soil physical and chemical properties measured before the experiment (February 2010).

Soil depth (cm)	Soil properties								
	Sand (%)	Silt (%)	Clay (%)	SP[a] (%)	CEC[b] (meq100 g^{-1})	K[c] (mg kg^{-1})	P[d] (mg kg^{-1})	$CaCO_3$ (%)	O.M.[e] (%)
0-30	46.0±3.3	26.0±0.8	28.0±3.4	48.2±3.0	27.7±3.7	53.2±4.2	12.6±2.8	21.8±2.5	0.56±0.03
30-60	43.8±5.0	26.8±2.3	29.4±5.5	47.2±6.0	26.9±4.8	43.2±2.6	6.5±1.5	25.2±4.1	0.45±0.04
60-90	43.4±7.6	23.6±1.2	33.0±6.8	47.6±6.0	28.0±5.4	45.9±2.6	3.7±0.5	25.4±4.6	0.39±0.03

Numbers are mean values of five sampling locations ± standard error of the mean.

[a] Saturation percentage.

[b] Cation exchange capacity, determined with sodium acetate extraction.

[c] $CaCl_2$ extraction.

[d] Olsen bicarbonate extraction.

[e] Organic matter content.

*The average soil saturated paste extract pH value was 7.6.

*Data was previously published in Haberman et al. (2019).
