# Supplementary material for: Long-Term Impact of Phosphorous Fertilization on Yield and Alternate Bearing in Intensive Irrigated Olive Cultivation
Source: Plants (Basel). 2021 Sep 1;10(9):1821. doi: 10.3390/plants10091821 (PMC8467881; doi:10.3390/plants10091821)
Supplement: Supplementary file 1 [file plants-10-01821-s001.zip › Tables S1-S8.pdf]

Table S1: Seasonal pruning weight.

P Fertilization ($kg\ ha^{-1}\ season^{-1}$)	Pruning weight ($kg\ tree^{-1}\ season^{-1}$)				
	2012	2013	2014	2015	2012-2015 mean
0	26.2±2.6 a	14.3±1.3 b	58.9±5.3 a	25.0±3.0 b	31.1±1.9 a
35	25.1±3.2 a	22.2±3.3 a	50.7±4.8 a	38.1±5.3 a	34.0±2.5 a

Numbers are mean values of 14 replicates (trees) ± standard error of the mean. Different letters indicate a statistically significant difference between P fertilization levels in the specified season ($P \leq 0.05$).

Table S2: Seasonal flowering intensity.

P Fertilization ($kg\ ha^{-1}\ season^{-1}$)	Flowering intensity (index)					
	2012	2014	2015	2016	2017	2014-2017 mean
0	3.4±0.27 b	1.0±0.43 b	4.2±0.43 a	2.5±0.39 b	4.5±0.27 a	3.05±0.07 b
35	4.5±0.14 a	3.3±0.41 a	2.9±0.53 a	3.8±0.33 a	3.5±0.40 b	3.34±0.11 a

Numbers are mean values of 14 replicates (trees) ± standard error of the mean. Different letters indicate a statistically significant difference between P fertilization levels in the specified season ($P \leq 0.05$).

Table S3: Seasonal inflorescences initiation rate.

P Fertilization ($kg\ ha^{-1}\ season^{-1}$)	Inflorescences initiation (%)		
	2014	2015	2014-2015 mean
0	16.6±6.3 b	56.0±6.8 a	36.3±2.4 b
35	55.0±6.4 a	34.3±7.3 b	44.5±3.2 a

Numbers are mean values of 14 replicates (trees) ± standard error of the mean. Different letters indicate a statistically significant difference between P fertilization levels in the specified season ($P \leq 0.05$).

Table S4: Seasonal single fruit weight.

P Fertilization ($kg\ ha^{-1}\ season^{-1}$)	Single fruit weight (g)						
	2011	2012	2013	2014	2015	2016	2013-2016 mean
0	2.4±0.13 b	3.1±0.19 a	1.9±0.18 b	3.1±0.22 a	2.6±0.07 a	2.6±0.31 a	2.66±0.11 a
35	3.0±0.24 a	3.1±0.18 a	2.6±0.19 a	2.7±0.18 a	2.6±0.10 a	1.3±0.19 b	2.42±0.09 a

Numbers are mean values of 14 replicates (trees) ± standard error of the mean. Different letters indicate a statistically significant difference between P fertilization levels in the specified season ($P \leq 0.05$).

Table S5: Seasonal fruit oil content.

P Fertilization (kg ha^{-1} season^{-1})	Oil content (%)							
	2010	2011	2012	2013	2014	2015	2016	2013-2016 mean
0	27.2±0.6 a	19.4±0.5 a	23.8±0.6 a	25.9±0.7 a	18.3±0.7 a	15.6±0.5 a	20.5±0.8 b	19.2±0.5 a
35	25.7±1.1 a	20.2±0.2 a	19.9±1.0 b	22.3±0.7 b	16.5±0.9 a	16.2±0.7 a	24.9±0.6 a	19.6±0.6 a

Numbers are mean values of 14 replicates (trees) ± standard error of the mean. Different letters indicate a statistically significant difference between P fertilization levels in the specified season ($P \leq 0.05$).

Table S6: Seasonal number of fruit per tree.

P Fertilization (kg ha^{-1} season^{-1})	Fruit per tree (g)						
	2011	2012	2013	2014	2015	2016	2013-2016 mean
0	21197±1963 a	10772±2386 a	25820±3468 a	4281±2006 b	16486±2303 a	10657±4441 b	14,153±825 b
35	16442±2312 a	14599±1700 a	19706±4101 a	16665±3631 a	10227±2804 a	32147±5692 a	19,813±1141a

Numbers are mean values of 14 replicates (trees) ± standard error of the mean. Different letters indicate a statistically significant difference between P fertilization levels in the specified season ($P \leq 0.05$).

Table S7: Seasonal fruit yield.

P Fertilization (kg ha^{-1} season^{-1})	Fruit yield (kg tree^{-1} season^{-1})							
	2010	2011	2012	2013	2014	2015	2016	2013-2016 mean
0	45.9±1.9 a	48.2±2.9 a	31.3±4.6 a	43.9±4.0 a	10.8±4.6 b	41.4±5.7 a	11.9±4.8 b	27.2±1.1 b
35	49.5±2.4 a	42.7±4.6 a	41.8±3.2 a	41.9±6.5 a	37.6±6.4 a	25.0±6.4 a	32.7±5.3 a	34.0±1.6 a

Numbers are mean values of 14 replicates (trees) ± standard error of the mean. Different letters indicate a statistically significant difference between P fertilization levels in the specified season ($P \leq 0.05$).

Table S8: Seasonal estimated oil yields.

P Fertilization (kg ha^{-1} season^{-1})	Oil yield (kg tree^{-1} season^{-1})							
	2010	2011	2012	2013	2014	2015	2016	2013-2016 mean
0	10.6±0.4 a	7.6±0.7 a	6.1±0.7 a	9.6±0.9 a	1.6±0.6 b	5.3±0.7 a	2.1±0.8 b	4.6±0.2 b
35	10.7±0.5 a	7.3±0.8 a	6.9±0.5a	7.8±1.3 a	5.1±0.9 a	3.6±1.0 a	6.8±1.1 a	5.6±0.4 a

Numbers are mean values of 14 replicates (trees) ± standard error of the mean. Different letters indicate a statistically significant difference between P fertilization levels in the specified season ($P \leq 0.05$).
