# Supplementary material for: Long-Term Impact of Phosphorous Fertilization on Yield and Alternate Bearing in Intensive Irrigated Olive Cultivation
Source: Plants (Basel). 2021 Sep 1;10(9):1821. doi: 10.3390/plants10091821 (PMC8467881; doi:10.3390/plants10091821)
Supplement: Supplementary file 1 [file plants-10-01821-s001.zip › Figure S1_Leaf N and K content.pdf]

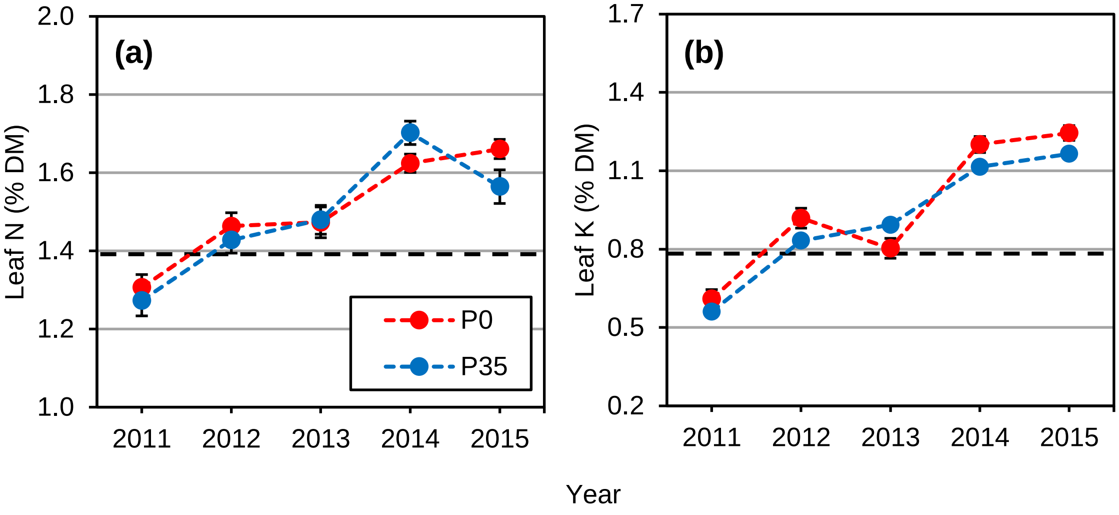

Figure S1. Effect of P fertilization on leaf N and K content as percent of dry mass (DM). Experiment plots were fertilized either with an annual amount of 35 kg P ha^{-1} (P35) or without P (P0). (a) Leaf N content. (b) Leaf K content. Numbers are mean values of 14 replicates (trees) ± standard error of the mean (bars). The horizontal dashed line indicates the putative deficiency-sufficiency threshold for N (a) and K (b) in leaves.
